# Association between retinal thickness and β-amyloid brain accumulation in individuals with subjective cognitive decline: Fundació ACE Healthy Brain Initiative

**DOI:** 10.1186/s13195-020-00602-9

**Published:** 2020-03-31

**Authors:** Marta Marquié, Sergi Valero, Miguel Castilla-Marti, Joan Martínez, Octavio Rodríguez-Gómez, Ángela Sanabria, Juan Pablo Tartari, Gemma C. Monté-Rubio, Oscar Sotolongo-Grau, Montserrat Alegret, Alba Pérez-Cordón, Natalia Roberto, Itziar de Rojas, Sonia Moreno-Grau, Laura Montrreal, Isabel Hernández, Maitee Rosende-Roca, Ana Mauleón, Liliana Vargas, Carla Abdelnour, Silvia Gil, Ester Esteban-De Antonio, Ana Espinosa, Gemma Ortega, Francisco Lomeña, Javier Pavia, Assumpta Vivas, Miguel Ángel Tejero, Marta Gómez-Chiari, Rafael Simó, Andreea Ciudin, Cristina Hernández, Adelina Orellana, Alba Benaque, Agustín Ruiz, Lluís Tárraga, Mercè Boada, on behalf of the FACEHBI study group, N. Aguilera, M. Berthier, M. Buendia, S. Bullich, F. Campos, P. Cañabate, C. Cuevas, A. Gailhajanet, J. Giménez, R. Gismondi, M. Guitart, M. Ibarria, A. Lafuente, E. Martín, M. Moreno, A. B. Nogales, L. Núñez, A. Páez, A. Pancho, E. Pelejà, V. Pérez-Grijalba, P. Pesini, S. Preckler, J. Romero, S. Ruiz, M. Sarasa, M. Torres

**Affiliations:** 1 Research Center and Memory Clinic, Fundació ACE Institut Català de Neurociències Aplicades - Universitat Internacional de Catalunya (UIC), Gran Via Carles III, 85 bis, 08028 Barcelona, Spain; 2 Centro de Investigación Biomédica en Red de Enfermedades Neurodegenerativas (CIBERNED), Instituto de Salud Carlos III, Madrid, Spain; 3 Clínica Oftalmológica Dr. Castilla, Barcelona, Spain; 4 Department of Ophthalmology, Hospital del Mar and Hospital de l’Esperança - Parc de Salut Mar, Barcelona, Spain; 5 Department of Radiology, Hospital Clínic i Provincial de Barcelona, Universitat de Barcelona, Barcelona, Spain; 6 Department of Diagnostic Imaging, Clínica Corachan, Barcelona, Spain; 7 Centro de Investigación Biomédica en Red de Diabetes y Enfermedades Metabólicas Asociadas (CIBERDEM), Instituto de Salud Carlos III, Madrid, Spain; 8 Institut de Recerca Vall d’Hebron, Universitat Autònoma de Barcelona (VHIR-UAB), Barcelona, Spain

**Keywords:** Optical coherence tomography, Retinal thickness, Subjective cognitive decline, β-Amyloid, Florbetaben, Positron emission tomography

## Abstract

**Background:**

Optical coherence tomography (OCT) of the retina is a fast and easily accessible tool for the quantification of retinal structural measurements. Multiple studies show that patients with Alzheimer’s disease (AD) exhibit thinning in several retinal layers compared to age-matched controls. Subjective cognitive decline (SCD) has been proposed as a risk factor for progression to AD. There is little data about retinal changes in preclinical AD and their correlation with amyloid-β (Aβ) uptake.

**Aims:**

We investigated the association of retinal thickness quantified by OCT with Aβ accumulation and conversion to mild cognitive impairment (MCI) over 24 months in individuals with SCD.

**Methods:**

One hundred twenty-nine individuals with SCD enrolled in Fundació ACE Healthy Brain Initiative underwent comprehensive neuropsychological testing, OCT scan of the retina and florbetaben (FBB) positron emission tomography (PET) at baseline (v0) and after 24 months (v2). We assessed the association of sixteen retinal thickness measurements at baseline with FBB-PET status (+/−) and global standardize uptake value ratio (SUVR) as a continuous measure at v0 and v2 and their predictive value on clinical status change (conversion to mild cognitive impairment (MCI)) at v2.

**Results:**

Mean age of the sample was 64.72 ± 7.27 years; 62.8% were females. Fifteen participants were classified as FBB-PET+ at baseline and 22 at v2. Every 1 μm of increased thickness in the inner nasal macular region conferred 8% and 6% higher probability of presenting a FBB-PET+ status at v0 (OR = 1.08, 95% CI = 1.02–1.14, *p* = 0.007) and v2 (OR = 1.06, 95% CI = 1.02–1.11, *p* = 0.004), respectively. Inner nasal macular thickness also positively correlated with global SUVR (at v0: *β* = 0.23, *p* = 0.004; at v2: *β* = 0.26, *p* = 0.001). No retinal measurements were associated to conversion to MCI over 24 months.

**Conclusions:**

Subtle retinal thickness changes in the macular region are already present in SCD and correlate with Aβ uptake.

## Introduction

In the past few decades, there has been an increase of the global prevalence of dementia that is threatening the sustainability of healthcare systems worldwide. Alzheimer’s disease (AD), responsible for 60–70% of all dementia cases, is a neurodegenerative condition that progressively and irreversibly impairs cognition and results in a complete loss of autonomy [1]. AD is the only disorder among the 10 principal causes of mortality still with no treatment or prevention [2]. Most AD cases are diagnosed once cognitive decline is already significant, but it is known that identifying the disease at earlier stages would result in cost savings and health benefits for patients, allowing them to modify their lifestyle, enrol in clinical trials, access programs of cognitive stimulation and social resources and make decisions about their future care. The disappointing results of clinical trials testing drugs against AD indicate that these interventions were implemented too late in the disease course and acting on an earlier stage would increase the chances of success [3]. Consequently, there is an urgent need to develop novel sensitive and specific biomarkers for the early identification of asymptomatic individuals at high risk for developing AD dementia before irreversible damage to the brain has been established and cognitive decline arises. In this regard, individuals with subjective cognitive decline (SCD) are a convenient target population for research studies on preclinical AD [4]. SCD refers to the self-perception of cognitive problems, including memory loss, without impairment on standardized cognitive tests [4]. Longitudinal studies have shown that elderly individuals with SCD have an increased risk of progression to cognitive impairment and dementia [5], more functional deficits [6] and higher prevalence of post-mortem AD pathology [7]. All these data point to SCD being the earliest clinical detectable point of the AD clinical continuum.

Brain accumulation of amyloid-β (Aβ) plaques and tau-containing neurofibrillary tangles are the main pathophysiological hallmarks of AD, along with neuronal and synaptic loss, inflammation and vascular pathology [8]. Currently, it is possible to quantify in vivo the Aβ burden decades before cognitive symptoms arise using positron emission tomography (PET) imaging of the brain with tracers against the amyloid protein [9] or by measuring Aβ_1-42_ and Aβ_1-40_ levels in the cerebrospinal fluid [10]. These tests have a good accuracy but are either quite expensive, invasive or not easily available in most healthcare centres, thus not useful for population screening [11].

The development of novel sensitive and specific biomarkers is currently one of the main goals in the AD research field. In this sense, optical coherence tomography (OCT) of the retina is a fast, inexpensive, non-invasive and easily accessible tool used to diagnose and monitor ocular pathologies such as diabetic retinopathy, open-angle glaucoma and age-related macular degeneration [12]. Embryologically, the retina and optic nerve expand from the diencephalon and are considered to be part of the central nervous system [13]. The retina shares many structural and functional features with the brain, such as the presence of neurons and glial cells, endothelial cells that form both the blood-brain and blood-retinal barriers [14], and axons of the optic nerve that connect the retina to the brain directly [15]. Thus, the retina is viewed as “a window to the brain” and is an attractive proxy to study brain biomarkers. In the past few years, OCT retinal structural changes have been demonstrated in several neurological diseases such as optic neuritis, multiple sclerosis, Parkinson’s disease and also AD [16]. Degeneration of the retinal nerve fiber layer (RNFL) and the ganglion cell layer (GCL) [17] along with the presence of Aβ plaques [18] has been observed in AD post-mortem tissue. Several studies have shown that patients with AD dementia and mild cognitive impairment (MCI) present with significant RNFL and GCL thinning measured by OCT of the retina compared to healthy individuals [19], although these changes are non-AD specific and have also been observed in other degenerative disorders such as chronic glaucoma. There is little data about retinal structural changes in preclinical AD and their relation with AD biomarkers.

In the present study, we aimed to determine whether baseline retinal structural measurements quantified by OCT were associated with Aβ deposition quantified by florbetaben (FBB) PET and clinical changes over 24 months in a cohort of individuals with SCD.

## Methods

### Study participants

Participants were selected among 200 individuals with SCD enrolled in *Fundació ACE Healthy Brain Initiative* (FACEHBI), a longitudinal observational study with the goal of investigating the pathophysiology of preclinical AD and the role of SCD as a risk marker for the future development of cognitive impairment [20]. FACEHBI participants were recruited from two different settings: Fundació ACE’s Memory Clinic [21] and the Open House Initiative (OHI) [22]. The OHI is a community-based engagement program that assesses cognitive status in individuals over 55 years for free and without the need of a physician’s referral. At baseline (v0) all participants underwent neurological and cognitive examinations, including the neuropsychological battery of Fundació ACE (N-BACE) [23, 24] and the Spanish version of the Face-Name Associative Memory Exam (S-FNAME) [25, 26], a set of self-administered questionnaires and a battery of multimodal biomarkers that included FBB-PET, brain magnetic resonance (MR), apolipoprotein E (APOE) genotyping and OCT scan of the retina.

FACEHBI inclusion criteria were as follows: age > 49 years, education level of at least elementary school (6 years of formal education), score ≥ 8 on the Spanish Modified Questionnaire of Memory Failures in Everyday (MFE-30) [27], score ≥ 27 on the Spanish version of the Mini-Mental State Examination (MMSE) [28, 29], a strictly normal performance on the N-BACE [23, 24], Clinical Dementia Rating Score (CDR) [30] of 0 and score < 11 on the Spanish version of the Hospital Anxiety and Depression (HAD) Scale [31]. Exclusion criteria were impairment in activities of daily living, presence of psychiatric diagnosis, history of alcoholism or epilepsy, renal or liver failure, and severe auditory or visual abnormalities.

After 24 months (v2), FACEHBI participants were reassessed using the same neuropsychological battery. A diagnosis of MCI was established when any of the N-BACE test scores was impaired according to the published cut-offs [23].

### Neuro-ophthalmological examination

At baseline, all participants underwent a neuro-ophthalmological evaluation that lasted about 20 min and was performed by an optometrist. It comprised (1) anamnesis about past ophthalmological diseases and treatments, (2) monocular visual acuity (VA) assessment using a pinhole occluder and the Early Treatment of Diabetic Retinopathy Study (ETDRS) chart [32], (3) intraocular pressure (IOP) measurement by Icare tonometry [33] and (4) OCT of the retina scan. Reduced visual acuity was defined as a decimal scale score ≤ 0.4 (equivalent to standard logMAR 6/15) using the ETDRS chart [32]. High IOP was defined as ≥ 24 mmHg using Icare Tonometry [33].

### Optical coherence tomography

At baseline, participants were imaged with a 3D-OCT Maestro®, Fast Map software version 8.40 (Topcon Co., Tokyo, Japan). No pupil dilation was performed. OCT of the retina capture was combined with a real colour fundus picture obtained through an internal camera.

Retinal layer segmentation was performed using the Topcon Advanced Boundary Segmentation TM (TABS) algorithm as part of the Fast Map software [34].

**Fig. 1:**
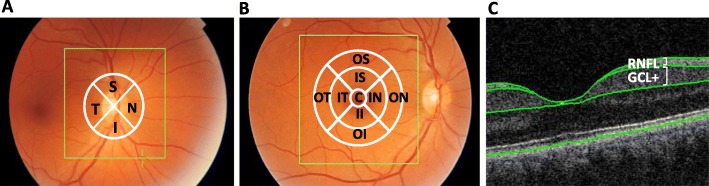
OCT of the retina imaging protocols. The three OCT retinal imaging protocols used for the analyses are depicted: **a** peripapillary RNFL, **b** macular ETDRS and **c** macular multilayer. Abbreviations: C centre, ETDRS Early Treatment Diabetic Retinopathy Study, GCL+ ganglion cell layer complex, I inferior, II inner inferior, IN inner nasal, IS inner superior, IT inner temporal, N nasal, RNFL retinal nerve fiber layer, OCT optical coherence tomography, OI outer inferior, ON outer nasal, OS outer superior, OT outer temporal, S superior, T temporal

Three different OCT of the retina protocols were selected for the analyses: *peripapillary RNFL*, *macular ETDRS* and *macular multilayer* (Fig. 1). The *peripapillary RNFL protocol* was performed using a circle scan around the optic disk and included five parameters: “total” refers to the average quantification of the whole region (360° measurement), “temporal” to the temporal quadrant thickness (316–45°), “superior” to the superior quadrant thickness (46–135°), “nasal” to the nasal quadrant thickness (136–225°) and “inferior” to the inferior quadrant thickness (226–315°). The *macular ETDRS protocol* was obtained scanning a 6 × 6 mm area that was divided in three regions: the “centre” region included the innermost 1-mm ring, the “inner” region included the ring ranging from 1 to 3 mm and the “outer” region included the ring ranging from 3 to 6 mm. Whole retinal thickness measurements from nine macular sub-regions (centre, inner temporal, inner superior, inner nasal, inner inferior, outer temporal, outer superior, outer nasal and outer inferior) were obtained in the macular ETDRS protocol. Lastly, the *macular multilayer protocol* included thickness quantifications of the GCL complex and RNFL in the whole macular region.

OCT-derived data from the right eye were analysed. OCT retinal images were reviewed by a consultant ophthalmologist expert in retinal pathology. OCT-related exclusion criteria were the following: lack of collaboration in the neuro-ophthalmological exam or OCT scan, OCT data obtained only from the left eye, presence of OCT artefacts and diseases that could affect retinal thickness (e.g. open-angle glaucoma and other neuropathies, maculopathies, prior retinal surgery, intraocular pressure [IOP] ≥ 24 mmHg, high myopia [< − 6Dp] or hyperopia [> + 6Dp] and optic nerve congenital abnormalities).

### FBB-PET image acquisition and processing

Previous to each FBB-PET scan, a MR was obtained on a Siemens© Magneton Aera (Erlangen, Germany) at Clínica Corachan, Barcelona. FBB-PET scans were acquired in a Siemens© Biograph molecular CT machine at the Radiology Department from the Hospital Clínic i Provincial in Barcelona. Four scans of 5 min each were obtained 90 min after the injection of 300 MBq of [F18]-FBB (Neuraceq©), administered as a single slow intravenous bolus (6 s/ml, total volume 10 ml). FBB was kindly provided by Life Molecular Imaging (previously Piramal). FBB data were co-registered to the MR-labelled data with the FSL 5.0 software package (https://fsl.fMRb.ox.ac.uk/fsl/fslwiki) and FreeSurfer (https://surfer.nmr.mgh.harvard.edu). A global cortical SUVR = 1.35 cut-off was selected to classify subjects into PET +/− status [35]. As brain amyloid uptake distribution among the study participants was not normally distributed, global SUVR quantifications were log transformed prior to their use in the analyses. MR and PET images were obtained in a 90-day window after clinical evaluations. More detailed information about MR and PET acquisition can be found elsewhere [20].

### Ethical considerations

A written consent was obtained from all participants prior to the enrolment in the study. The FACEHBI protocol received approval from the ethics committee of Hospital Clínic i Provincial in Barcelona, Spain (EudraCT number 2014-00079-38). The referral centre ethics committee approved the patient recruitment, and collection protocols were in accordance with ethical standards according to World Medical Association Declaration of Helsinki—Ethical Principles for Medical Research Involving Human Subjects.

### Statistical analysis

Data processing and analysis were conducted using SPSS v.24. Alpha level was set at 0.05.

Demographical differences between PET+/− and clinical groups were assessed using a two-tailed *T* test for quantitative variables and a chi-square test for qualitative ones, respectively.

Thicknesses of sixteen retinal regions at baseline were considered as predictors of PET status (+/−) at v0 and v2 and of clinical status (SCD vs MCI) at v2, separately. In order to detect the most significant regions and control for false positives, all analyses were executed in two steps.

Retinal thickness was analysed as predictor of PET status at v0. First, several logistic regression models were executed (one for each region), with FBB-PET status as the outcome. Age, gender, years of education, APOE ε4 status and OCT of the retina image quality were included as adjusting variables. A second step was carried out, including simultaneously those retinal measures that obtained a significant effect in the former step, including the same adjusting variables.

The same sixteen retinal measures, regression model, adjusting variables and two-step strategy were employed to analyse retinal thickness as predictor of PET status and clinical status at v2, separately.

All logistic models were reported as odds ratio (OR) with 95% confidence intervals, expressed as the probability of presenting a PET+ at v0 or v2 or converting to MCI at v2 per each 1-μm retinal thickness change, respectively.

Finally, we explored the association of PET expressed as a quantitative variable with retinal thickness. For that, several linear regression models were run using log-transformed FBB global SUVR as the outcome (one for each of the sixteen retinal measures as predictors), at v0 and v2 separately. The same adjusting variables were included. In a second step, those measures that were significant in the former step were analysed simultaneously, including again the adjusting variables.

In all these statistical analysis, the goal of the first univariate step was to identify potential discriminant factors (thickness of different retinal regions as predictors of PET and clinical status, separately) that would then compete among them in a second multivariate step, in order to avoid a large number of predictors in the latter. No specific Bonferroni cut-off was imposed in the first exploratory step, as this could have supposed the exclusion of significant relevant factors in the final analysis, which would have been “penalized” by high *p* significance values.

## Results

### Demographic characteristics

**Fig. 2:**
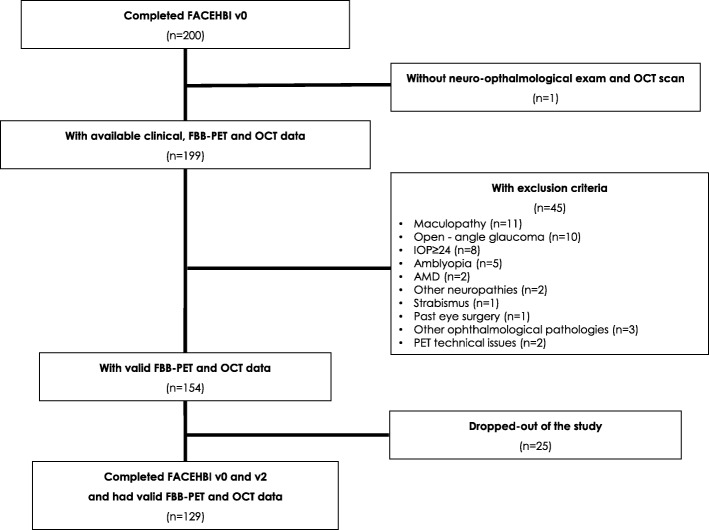
Study flow chart. Eligible FACEHBI participants and selection for the final study sample through inclusion and exclusion criteria. Abbreviations: AMD age-related macular degeneration, FACEHBI Fundació ACE Healthy Brain Initiative, FBB florbetaben, IOP intraocular pressure, OCT optical coherence tomography, PET positron emission tomography, v0 baseline visit, v2 2-year follow-up visit

Two hundred individuals with SCD enrolled in FACEHBI underwent the baseline visit (v0) between December 2014 and March 2016. All participants but one completed the OCT retinal scan. Forty-three individuals were excluded from the analysis due to eye-related pathologies that could potentially affect retinal thickness measurements. Specific causes for exclusion were as follows: previous diagnosis of maculopathy (*n* = 11) or open-angle glaucoma (*n* = 10), IOP ≥ 24 (*n* = 8), amblyopia (*n* = 5), AMD (*n* = 2), other neuropathies (*n* = 2), strabismus (*n* = 1), past eye surgery (*n* = 1) and others (*n* = 3). Two individuals were excluded due to FBB-PET technical reasons. Finally, 25 participants dropped out before v2. The study flow chart is depicted in Fig. 2. Those 71 participants excluded from the study were significantly older (67.36 ± 7.20 vs 64.72 ± 7.27, *p* = 0.01) and had lower MMSE scores (28.99 ± 99 vs 29.35, *p* = 0.008) than those comprising the final study sample. There were no statistical differences in years of education, FBB global SUVR, gender, APOE ε4 status and clinical status at v2 (*p* > 0.05) between excluded and included participants. No significant differences in retinal thickness were detected except in the inferior peripapillary RNFL region, where excluded participants showed significant thinning (122.17 ± 29.66 vs 132.74 ± 18.01, *p* = 0.008) (Additional file 1).

The final sample consisted of 129 SCD individuals with available OCT of the retina at baseline and FBB-PET at v0 and v2. Demographic characteristics of the cohort are detailed in Table 1. Mean age at v0 was 64.72 ± 7.27 years, 62.8% were female, education was 12.49 ± 3.94 years, MMSE score was 29.35 ± 0.88 and 24.8% of the participants were APOE ε4 carriers.

**Table 1:** Demographic characteristics of the study cohort

	PET status at v0			PET status at v2			Clinical status at v2		
	Aβ−	Aβ+	***p***	Aβ−	Aβ+	***p***	SCD	MCI	***p***
*n*	114	15	N/A	107	22	N/A	114	15	N/A
Age	64.22 ± 7.41	68.49 ± 4.76	0.03*	65.70 ± 7.29	70.82 ± 5.48	0.002*	65.76 ± 6.77	72.73. ± 8.12	< 0.001*
Females (%)	71 (62.3%)	10 (66.7%)	0.74	68 (63.55%)	13 (59.1%)	0.69	72 (63.16%)	9 (60%)	0.85
Years of education	12.58 ± 3.94	11.80 ± 3.99	0.48	12.56 ± 3.91	12.14 ± 4.16	0.65	12.80 ± 3.86	10.07 ± 3.83	0.01*
MMSE	29.32 ± 0.90	29.53 ± 0.74	0.39	29.22 ± 1.03	29.11 ± 1.32	0.72	29.33 ± 0.88	28.25 ± 1.82	0.06
APOE ε4+ (%)	21 (18.4%)	11 (73.7%)	< 0.001*	21 (19.6%)	11 (50%)	0.003*	24 (21.05%)	8 (53.3%)	0.007*

A *T* test was used to analyse differences on age, years of education and MMSE between groups. A Chi-square test was employed to analyse differences on the distribution of females and APOE genotype between groups

*Statistical significance was set-up at *p* < 0.05

*Abbreviations*: *Aβ* amyloid-β, *APOE* apolipoprotein E, *MMSE* Mini-Mental State Examination, *PET* positron emission tomography, *MCI* mild cognitive impairment, *SCD* subjective cognitive decline, *v0* baseline visit, *v2* 2-year follow-up visit

**Fig. 3:**
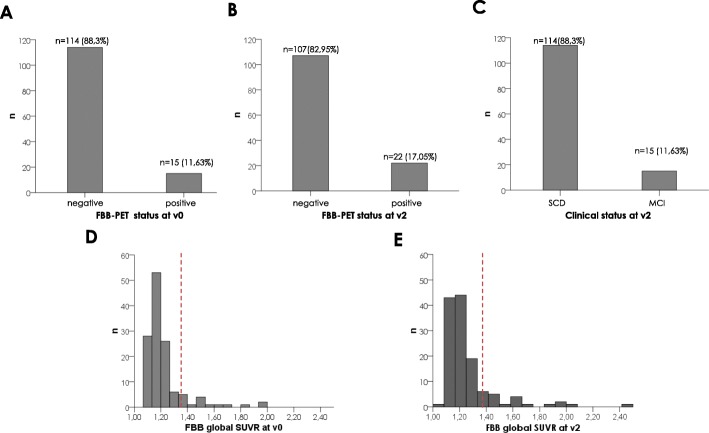
FBB-PET and clinical status of the study participants. The red dashed vertical line in **d** and **e** represents the global SUVR cut-off value of 1.35 [35]. Abbreviations: FBB florbetaben, PET positron emission tomography, SUVR standardized uptake value ratio, v0 baseline visit, v2 2-year follow-up visit

At v0, SCD participants were classified according to a FBB global SUVR cut-off = 1.35 as Aβ− (*n* = 114, 88.37%, mean global SUVR = 1.17 ± 0.05) and Aβ+ (*n* = 15, 11.63%, mean global SUVR = 1.57 ± 0.20) (Fig. 3a, d). Aβ+ participants were significantly older (68.49 ± 4.76 vs 64.22 ± 7.41, *p* = 0.03) and more frequently APOE ε4 carriers (73.7% vs 18.4%, *p* < 0.001) than those Aβ−, while there were no differences in gender, education and MMSE scores (Table 1).

At v2, 7 participants (6.14%) converted to an Aβ+ status. The sample was divided into Aβ− (*n* = 107, 82.95%, mean global SUVR = 1.19 ± 0.06) and Aβ+ (*n* = 22, 17.05%, mean global SUVR = 1.61 ± 0.28) groups (Fig. 3b, e). Aβ+ participants were significantly older (70.82 ± 5.48 vs 65.70 ± 7.29, *p* = 0.002) and more frequently APOE ε4 carriers (50% vs 19.4%, *p* = 0.003) than those who were Aβ−, while there were no differences in gender, education and MMSE scores (Table 1).

At v2, 15 participants converted from SCD to MCI according to their neuropsychological profile (Fig. 3c). Eight of them had an amnestic subtype and 7 a non-amnestic subtype MCI. Those who converted were significantly older (72.73 ± 8.12 vs 65.76 ± 6.77, *p* < 0.001), had less years of education (10.07 ± 3.83 vs 12.80 ± 3.86, *p* = 0.01) and were more frequently APOE ε4 carriers (53.5% vs 21.05%, *p* = 0.007) than those who remained as SCD (Table 1). Not unexpectedly, there was not a perfect overlap between conversion to MCI and Aβ+ status: out of the 15 MCI converters, only 5 were Aβ+ at v0 and 6 at v2.

Regarding the study settings, 90 participants (69.77%) were recruited through the OHI and 39 (30.23%) through the Memory Clinic. Individuals from the latter group were significantly older (66.89 ± 7.99 vs 63.77 ± 6.76, *p* = 0.025) and had a higher risk of MCI conversion over 24 months (23.01% vs 6.67%, *p* = 0.008). FBB global SUVR differences were borderline significant, with participants from the Memory Clinic having higher SUVR (1.27 ± 0.23 vs 1.20 ± 0.10, *p* = 0.06). There were no differences in terms of gender, education, MMSE scores and APOE ε4 status (Additional file 2).

### Association of retinal thickness measurements with FBB-PET status

Correlations of retinal thickness among the sixteen regions selected are shown in Additional file 3. As expected, the within-region correlations were high, while correlations between different regions were low to moderate.

Thickness measurements from the sixteen retinal regions from the Aβ− and Aβ+ groups at v0 and v2 and from the SCD and MCI groups at v2, respectively, are depicted in Table 2. Raw data showed that Aβ+ participants had lower peripapillary RNFL thickness measures at v0, and this difference was amplified at v2. On the contrary, inner macular thickness appeared to be increased in Aβ+ individuals. No apparent group differences were observed in other retinal regions.

**Table 2:** Retinal thickness measurements by PET and clinical status

	PET status (+/−) at v0		PET status (+/−) at v2		Clinical status at v2	
	Aβ−	Aβ+	Aβ−	Aβ+	SCD	MCI
*n*	114	15	107	22	114	15
FBB global SUVR	1.17 ± 0.05	1.57 ± 0.20	1.19 ± 0.06	1.61 ± 0.28	1.24 ± 0.16	1.41 ± 0.39
Peripapillary RNFL						
Total	100.74 ± 11.84	96.90 ± 19.38	101.27 ± 11.33	95.55 ± 18.40	100.123 ± 13.24	101.56 ± 10.24
Temporal	74.02 ± 10.67	74.23 ± 15.23	74.33 ± 10.60	72.64 ± 14.05	73.84 ± 11.20	75.58 ± 11.62
Superior	118.05 ± 19.75	118.27 ± 18.64	118.85 ± 18.46	114.14 ± 24.59	118.05 ± 19.91	118.26 ± 17.31
Nasal	77.57 ± 16.28	74.24 ± 17.82	77.96 ± 15.75	73.45 ± 19.37	77.16 ± 16.77	77.41 ± 14.02
Inferior	133.28 ± 17.62	128.62 ± 21.0	133.89 ± 17.82	127.14 ± 18.31	132.44 ± 18.50	134.97 ± 14.04
Image quality	47.41 ± 7.42	46.87 ± 7.42	47.52 ± 7.48	46.50 ± 7.02	47.99 ± 7.12	42.44 ± 7.78
ETDRS macula						
Centre	249.67 ± 21.63	259.91 ± 23.14	249.07 ± 21.56	259.59 ± 22.32	250.03 ± 22.09	257.18 ± 20.63
Inner temporal	300.01 ± 15.72	305.52 ± 15.55	299.56 ± 15.15	305.91 ± 17.76	300.10 ± 15.63	304.80 ± 16.48
Inner superior	312.32 ± 14.54	316.96 ± 18.05	311.74 ± 14.11	318.30 ± 18.07	312.43 ± 14.83	316.14 ± 16.24
Inner nasal	313.41 ± 14.26	321.38 ± 17.62	312.81 ± 13.64	321.71 ± 18.29	313.80 ± 14.89	318.35 ± 14.25
Inner inferior	309.64 ± 14.37	316.04 ± 18.48	309.14 ± 13.87	316.46 ± 18.60	309.64 ± 14.49	316.05 ± 17.70
Outer temporal	254.28 ± 14.26	250.72 ± 15.70	254.22 ± 14.10	252.15 ± 16.11	253.75 ± 14.00	254.75 ± 17.81
Outer superior	269.66 ± 12.92	266.47 ± 16.49	269.48 ± 12.90	268.36 ± 15.65	269.28 ± 12.84	269.34 ± 17.27
Outer nasal	285.87 ± 14.42	288.58 ± 18.38	285.80 ± 14.03	288.05 ± 18.71	285.65 ± 14.16	290.20 ± 19.58
Outer inferior	258.75 ± 13.50	260.79 ± 20.32	258.85 ± 13.69	259.62 ± 17.67	258.43 ± 13.45	263.23 ± 20.12
Image quality	47.60 ± 6.94	48.68 ± 6.40	47.76 ± 7.01	47.55 ± 6.26	48.53 ± 6.36	41.63 ± 7.70
Macular multilayer						
GCL+	64.32 ± 4.85	64.47 ± 4.54	64.36 ± 4.79	64.22 ± 4.94	64.42 ± 4.60	63.68 ± 6.25
RNFL	38.02 ± 4.39	39.62 ± 4.96	38.06 ± 4.42	39.93 ± 4.74	38.17 ± 4.37	38.41 ± 5.31
Image quality	47.06 ± 6.94	50.04 ± 5.95	47.19 ± 7.02	48.48 ± 6.17	47.62 ± 6.82	45.80 ± 7.33

Data were expressed as mean ± standard deviation. OCT retinal measurements were expressed as thickness (μm) except for image quality

*Abbreviations*: *Aβ* amyloid-β, *FBB* florbetaben, *ETDRS* Early Treatment Diabetic Retinopathy Study, *GCL* ganglion cell layer, *MCI* mild cognitive impairment, *PET* positron emission tomography, *RNFL* retinal nerve fiber layer, *SCD* subjective cognitive decline, *SUVR* standardized uptake value ratio, *v0* baseline visit, *v2* follow-up visit at 2 years

The logistic regression model showed, in the initial step, that five macular sub-areas of the ETDRS chart had an independent significant effect on FBB-PET status at v0 (centre: OR = 1.04, *p* = 0.03; inner temporal: OR = 1.06, *p* = 0.03; inner superior: OR = 1.05, *p* = 0.047; inner nasal: OR = 1.08, *p* = 0.007; and inner inferior: OR = 1.05, *p* = 0.04) and v2 (centre: OR = 1.03, *p* = 0.03; inner temporal: OR = 1.05, *p* = 0.03; inner superior OR = 1.05, *p* = 0.02; inner nasal: OR = 1.06, *p* = 0.004; and inner inferior: OR = 1.05, *p* = 0.02) (Table 3).

**Table 3:** Logistic regression model output of the association of retinal thickness measurements at baseline with PET status at v0 and v2 and clinical status change over 24 months

Retinal regions	PET status at v0				PET status at v2				Clinical status at v2			
	Wald	OR	95% CI	***p***	Wald	OR	95% CI	***p***	Wald	OR	95% CI	***p***
Peripapillary RNFL												
Total	0.69	0.98	0.94–1.03	0.39	2.76	0.97	0.94–1.01	0.10	2.38	1.06	0.98–1.14	0.12
Temporal	0.60	1.03	0.96–1.10	0.44	0.14	0.99	0.94–1.04	0.71	2.81	1.06	0.99–1.14	0.09
Superior	0.37	1.01	0.98–1.05	0.54	0.26	0.99	0.97–1.02	0.61	1.04	1.02	0.98–0.16	0.31
Nasal	1.19	0.98	0.95–1.02	0.28	1.93	0.98	0.95–1.00	0.17	0.03	1.00	0.96–1.05	0.87
Inferior	0.13	0.99	0.96–1.03	0.72	1.18	0.98	0.96–1.01	0.28	3.08	1.04	1.00–1.09	0.08
ETDRS macula												
Centre	4.93	1.04	1.01–1.08	0.03*	4.98	1.03	1.00–1.06	0.03*	2.11	1.03	0.99–1.06	0.15
Inner temporal	4.88	1.06	1.01–1.12	0.03*	4.82	1.05	1.01–1.09	0.03*	1.48	1.03	0.98–1.08	0.23
Inner superior	3.93	1.05	1.01–1.10	0.047*	5.54	1.05	1.00–1.09	0.02*	1.48	1.03	0.98–1.08	0.22
Inner nasal	7.20	1.08	1.02–1.14	0.007*	8.39	1.06	1.02–1.10	0.004*	1.33	1.03	0.98–1.07	0.25
Inner inferior	4.14	1.05	1.00–1.10	0.04*	5.60	1.05	1.01–1.09	0.02*	2.34	1.04	0.99–1.09	0.13
Outer temporal	0.52	1.02	0.97–1.07	0.47	0.22	1.01	0.97–1.05	0.64	1.26	1.03	0.98–1.09	0.26
Outer superior	0.11	1.00	0.96–1.06	0.74	0.21	1.01	0.97–1.05	0.65	0.73	1.02	0.97–1.08	0.39
Outer nasal	2.47	1.04	0.99–1.08	0.12	1.70	1.02	0.99–1.06	0.19	2.60	1.04	0.99–1.08	0.11
Outer inferior	2.87	1.04	0.99–1.09	0.09	1.52	1.02	0.99–106	0.22	3.63	1.05	1.00–1.11	0.06
Macular multilayer												
GCL+	2.22	1.12	0.96–1.31	0.14	1.65	1.08	0.96–1.21	0.20	0.76	1.07	0.92–1.24	0.38
RNFL	2.35	1.12	0.97–1.30	0.13	1.05	1.06	0.95–1.19	0.31	0.32	1.04	0.91–1.20	0.58

A 2-step logistic regression model was executed for each of the sixteen retinal thickness measures as predictors of PET status (+/−) at v0 and v2 and clinical status (SCD vs MCI) at v2, separately, and including age, gender, education, APOE ε4 status and OCT image quality as adjusting variables

*Statistical significance was set-up at *p* < 0.05

*Abbreviations*: *APOE* apolipoprotein E, *CI* confidence interval, *ETDRS* Early Treatment Diabetic Retinopathy Study, *GCL* ganglion cell layer, *OR* odds ratio, *PET* positron emission tomography, *RNFL* retinal nerve fiber layer, *v0* baseline visit, *v2* follow-up visit at 2 years

**Fig. 4:**
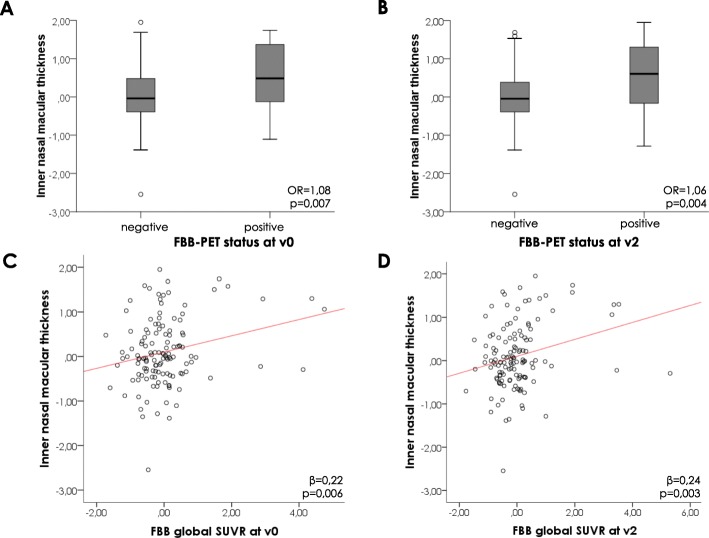
Relationship between inner nasal macular thickness and FBB-PET. Inner nasal macular thickness differences according to the participants’ FBB-PET status (+/−) at v0 (**a**) and at v2 (**b**). Correlation between inner macular thickness and FBB global SUVR at v0 (**c**) and at v2 (**d**). Both inner nasal macular thickness and FBB global SUVR are expressed as standardized scores after adjustment by age, gender, education, APOE ε4 status and OCT image quality. Abbreviations: APOE apolipoprotein E, FBB Flobetaben, PET positron emission tomography, SUVR standardized uptake value ratio, v0 baseline visit, v2 2-year follow-up visit

In a subsequent multivariate analysis, those five macular regions selected in the former step were analysed together competing with education, gender, age, APOE ε4 status and OCT retinal image quality as adjusting covariates, at v0 and v2 separately. Only macular inner nasal thickness remained as significant predictor of FBB-PET status at both v0 and v2 (Table 4). The obtained model showed that every 1 μm of increased thickness in the inner nasal macular region conferred a 8% and 6% higher probability of presenting a PET+ at v0 and v2, respectively (at v0: OR = 1.08, *p* = 0.007; at v2: OR = 1.06, *p* = 0.004) (Fig. 4a, b).

**Table 4:** Logistic regression model output of the association of macular retinal thickness with PET status at v0 and v2

	PET status at v0				PET status at v2			
	Wald	***p***	OR	95% CI	Wald	***p***	OR	95% CI
Years of education	0.003	0.96	1.01	0.84–1.02	0.32	0.57	1.04	0.90–1.21
Gender	0.79	0.37	0.50	0.11–2.29	1.15	0.28	0.52	0.16–1.72
Age	7.00	0.008*	1.20	1.05–1.37	9.59	0.002*	1.17	1.06–1.29
APOE ε4 status	16.03	< 0.001*	32.06	5.87–175.25	10.26	0.001*	6.65	2.09–21.22
OCT retinal image quality	4.03	0.05	1.16	1.00–1.33	1.51	0.22	1.06	0.97–1.16
Inner nasal macular thickness	7.20	0.007*	1.08	1.02–1.14	8.39	0.004*	1.06	1.02–1.11

The five retinal regions that obtained a significant effect in the previous step of the logistic regression model (ETDRS macular centre, inner temporal, inner superior, inner nasal and inner inferior areas) were subsequently analysed together, including education, gender, age, APOE ε4 status and OCT retinal image quality as adjusting variables, separately for v0 and v2. Only inner nasal macular thickness remained as a significant predictor of PET status at v0 and v2, and the obtained model showed that increased thickness in this region at baseline conferred higher probability of a PET status both at v0 and v2

*Statistical significance was set-up at *p* < 0.05

*Abbreviations*: *APOE* apolipoprotein E, *CI* confidence interval, *ETDRS* Early Treatment Diabetic Retinopathy Study, *OCT* optical coherence tomography, *OR* odds ratio, *PET* positron emission tomography, *v0* baseline visit, *v2* follow-up visit at 2 years

### Association of retinal thickness measures with FBB global SUVR

A linear regression analysis was used to assess the association of sixteen selected retinal thickness measures with log-transformed FBB global SUVR at v0 and v2 respectively, including education, gender, age, APOE ε4 status and OCT retinal image quality as covariates (Table 5). The model identified that the thickness of four macular sub-regions was significantly and positively related to global SUVR at v0 (centre: *β* = 0.19, *p* = 0.02; inner nasal: *β* = 0.23, *p* = 0.004; inner inferior: *β* = 0.18, *p* = 0.03; and outer inferior: *β* = 0.18, *p* = 0.03) and five macular regions at v2 (centre: *β* = 0.18, *p* = 0.03; inner superior: *β* = 0.18, *p* = 0.03; inner nasal: *β* = 0.26, *p* = 0.001; inner inferior: *β* = 0.20, *p* = 0.01; and outer inferior: *β* = 0.19, *p* = 0.03).

**Table 5:** Linear regression analysis output of the association of retinal thickness at baseline with FBB global SUVR at v0 and v2

Retinal regions	FBB global SUVR at v0					FBB global SUVR at v2				
	***B***	95% CI	***t***	***β***	***p***	***B***	95% CI	***t***	***β***	***p***
Peripapillary RNFL										
Total	− 0.001	− 0.001–0.000	− 1.87	− 0.16	0.06	− 0.001	− 0.001–0.000	− 1.66	− 0.14	0.10
Temporal	0.000	− 0.001–0.001	− 1.28	− 0.11	0.20	0.000	− 0.001–0.000	− 1.03	− 0.09	0.31
Superior	0.000	0.000–0.000	− 0.27	− 0.02	0.79	0.000	0.000–0.000	− 0.12	− 0.12	0.91
Nasal	0.000	− 0.001–0.000	− 1.38	− 0.11	0.17	0.000	− 0.001–0.000	− 1.42	− 0.12	0.16
Inferior	0.000	0.000–0.000	− 0.31	− 0.03	0.76	0.000	− 0.001–0.000	− 0.40	− 0.03	0.69
ETDRS macula										
Centre	0.000	0.000–0.001	2.37	0.19	0.02*	0.000	0.000–0.001	2.17	0.18	0.03*
Inner temporal	0.000	0.000–0.001	1.86	0.15	0.07	0.001	0.000–0.001	1.95	0.16	0.05
Inner superior	0.001	0.000–0.001	1.76	0.14	0.08	0.001	0.000–0.001	2.18	0.18	0.03*
Inner nasal	0.001	0.000–0.001	2.95	0.23	0.004*	0.001	0.000–0.002	3.27	0.26	0.001*
Inner inferior	0.001	0.000–0.001	2.23	0.18	0.03*	0.001	0.000–0.001	2.55	0.20	0.01*
Outer temporal	0.000	0.000–0.001	0.62	0.05	0.54	0.000	− 0.001–0.001	0.37	0.03	0.71
Outer superior	0.000	0.000–0.001	0.54	0.05	0.59	0.000	0.000–0.001	0.82	0.07	0.41
Outer nasal	0.000	0.000–0.001	1.25	0.10	0.21	0.001	0.000–0.001	1.65	0.13	0.10
Outer inferior	0.001	0.000–0.001	2.22	0.18	0.03*	0.001	0.000–0.001	2.25	0.19	0.03*
Macular multilayer										
GCL+	0.001	− 0.001–0.003	1.05	0.09	0.30	0.001	− 0.001–0.003	0.86	0.08	0.39
RNFL	0.001	− 0.001–0.002	0.91	0.07	0.37	0.001	− 0.001–0.003	1.03	0.08	0.30

A linear regression model for each of the sixteen retinal thickness measures was executed using log-transformed FBB global SUVR as the outcome, for v0 and v2 separately. In all analysis age, gender, education, APOE ε4 status and OCT retinal image quality were included as covariates

*Statistical significance was set-up at *p* < 0.05

*Abbreviations*: *APOE* apolipoprotein E, *CI* confidence interval, *ETDRS* Early Treatment Diabetic Retinopathy Study, *FBB* florbetaben, *GCL* ganglion cell layer complex, *OCT* optical coherence tomography, *OR* odds ratio, *RNFL* retinal nerve fiber layer, *SUVR* standardized uptake value ratio, *v0* baseline visit, *v2* follow-up visit at 2 years

The macular regions selected in the former step were then analysed together competing with the same covariates (Table 6). Only inner nasal macular thickness remained significantly associated with FBB global SUVR. The model showed that increased thickness in the inner nasal macular region was associated with higher FBB global SUVR both at baseline and after 24 months (at v0: *β* = 0.23, *p* = 0.004; at v2: *β* = 0.26, *p* = 0.001) (Fig. 4c, d).

**Table 6:** Linear regression analysis output of the association of retinal thickness with FBB global SUVR at v0 and v2

	Log-transformed FBB global SUVR at v0					Log-transformed FBB global SUVR at v2				
	***B***	95% CI	***t***	***β***	***p***	***B***	95% CI	***t***	***β***	***p***
Years of education	0.001	− 0.001–0.003	1.04	0.09	0.30	0.001	− 0.001–0.003	0.81	0.07	0.42
Gender	0.000	− 0.016–0.016	− 0.04	− 0.002	0.98	0.001	− 0.019–0.021	0.10	0.008	0.92
Age	0.001	0.000–0.003	2.70	0.23	0.008*	0.002	0.001–0.004	3.30	0.28	0.004*
APOE ε4 status	0.044	0.027–0.061	5.16	0.41	< 0.001*	0.049	0.028–0.070	4.60	0.37	< 0.001*
OCT image quality	0.001	0.000–0.002	1.73	0.14	0.09	0.001	0.000–0.002	1.41	0.11	0.16
Inner nasal macular thickness	0.001	0.000–0.001	2.95	0.23	0.004*	0.001	0.000–0.002	3.27	0.26	0.001*

A second step of the linear regression model was executed with log-transformed FBB global SUVR as the outcome, including simultaneously the regions with a significant effect in the previous step (macular ETDRS centre, inner nasal, inner inferior and outer inferior areas for v0, and macular ETDRS centre, inner nasal, inner superior, inner inferior and outer inferior areas for v2) as predictors, separately. In all analysis, years of education, gender, age, APOE ε4 status and OCT retinal image quality were included as adjusting covariates. The output model showed that only retinal thickness in the inner nasal macular sub-region was positively associated with global SUVR, both at v0 and v2

*Statistical significance was set-up at *p* < 0.05

*Abbreviations*: *APOE* apolipoprotein E, *FBB* florbetaben, *CI* confidence interval, *ETDRS* Early Treatment Diabetic Retinopathy Study, *OCT* optical coherence tomography, *OR* odds ratio, *RNFL* retinal nerve fiber layer, *SUVR* standardized uptake value ratio, *v0* baseline visit, *v2* visit at 2 years

### Association of retinal thickness measurements with clinical status change at v2

Thickness measurements from the sixteen selected retinal regions divided by clinical status (SCD vs MCI) at v2 are shown in Table 2.

A logistic regression model was run to analyse the independent predictive value of the sixteen retinal thickness measures on the clinical status at v2 (Table 3). None of the retinal thickness measures analysed had a significant effect on conversion to MCI over 24 months.

## Discussion

In this study, we analysed the association of retinal structural measurements with Aβ brain accumulation in 129 individuals with SCD. Our data identified a significant thickening of the inner nasal macular region in very early AD stages (SCD Aβ+ individuals) and a positive association between thickness in this same region and FBB global SUVR. However, retinal thickness did not predict conversion to MCI over 24 months.

Our study cohort included 129 participants from the FACEHBI study with SCD who underwent FBB-PET and OCT of the retina scan at baseline and after 24 months. The diagnosis of SCD involves the self-perception of cognitive problems with a strictly normal performance on a comprehensive neuropsychological battery and preservation of autonomy in daily life activities [4]. At baseline, 15 participants were classified as Aβ+ using a FBB global SUVR cut-off = 1.35 [35]. As our cohort was comprised of SCD, which has been proposed as the earliest symptom of the AD continuum, and we aimed to identify the very initial stages of amyloid accumulation, we chose a liberal/low cut-off to divide the sample. In spite of this, the resulting 11.63% rate of Aβ positivity is slightly lower than those reported in other series [36–38]. Several reasons could account for this, including the mean age of the sample (our study participants were relatively young, while it is well known that amyloid positivity increases with age [39]), the definition of cognitive normality (our study used strict neuropsychological criteria, such that only one score below the established cut-offs in any single N-BACE [23] test precluded an individual to be classified as SCD) and finally the setting of the study (70% of our sample came from the OHI vs 30% from the Memory Clinic). A recent multicentric study highlighted that the risk of dementia is strongly increased in SCD individuals from a clinical setting but less in a community-based setting [40]. In line with that, our participants from the Memory Clinic were significantly older and had higher risk of converting to MCI and a trend to higher FBB global SUVR.

The main finding of the study is that structural retinal changes are already present in preclinical AD stages and can be detected by OCT. Our data showed that Aβ+ SCD participants exhibited a significant thickening of the inner nasal macular region at baseline even after controlling for age, gender, education, APOE ε4 status and OCT retinal image quality, both at baseline and after 24 months. Additionally, RNFL thinning in Aβ+ participants was observed at v2, although its difference compared to those Aβ− did not reach statistical significance.

Our results do not completely concur with those of similar studies. Of note, few groups have investigated the relationship between retinal thickness and Aβ accumulation in non-demented individuals and most articles included a relatively low number of participants, reported only cross-sectional data and considerably inconsistent findings regarding the regions and direction of retinal changes (thinning vs thickening) associated with Aβ deposition. In order to correctly interpret OCT of the retina literature results in the dementia field, it is important to have in mind that retinal thickness data obtained from different OCT devices are not readily interchangeable and it is relevant to know the average thickness and volumes obtained from the particular OCT device used in each study, which are known to be influenced by age, gender and ocular pathologies [41]. Additionally, two previous studies from our own group highlighted that OCT retinal image quality significantly differs among SCD, MCI and dementia groups and is an important predictor of OCT retinal image variability within these populations [42, 43]. Still, most studies in the dementia field do not include OCT retinal image quality as a covariate in the analysis, while our data show that it would be advisable to do so. Thus, comparing results from the literature should be done with caution.

Golzan et al. observed a slight GCL thinning in 28 AD dementia patients compared to 50 Aβ− controls but no differences with preclinical AD individuals [44]. O’Bryhim et al. detected an increase in the foveal avascular zone area and a reduction in the inner foveal thickness in an Aβ+ group (*n* = 14) compared to 16 controls [45]. Recently, van de Kreeke et al. did not detect differences in macular or RNFL thickness between 18 Aβ+ and 147 Aβ− healthy elderly monozygotic twins [46].

In line with our findings showing retinal thickening in the pre-dementia AD phase, Snyder et al. reported that the surface area of inclusion bodies increased as a function of amyloid burden in a group of 63 cognitively healthy individuals with a trend toward a selective volume increase in the inner plexiform layer in Aβ+ individuals (*n* = 10) compared to those Aβ− (*n* = 53) [47]. Ascaso et al. showed an increase of macular thickness and volume in 21 MCI patients compared to 18 AD and 41 healthy individuals [48]. In the twin study published by van de Kreet et al. reported above, a positive association between flutemetamol uptake and inner macular thickness was observed, although did not remain significant after multiple testing correction [46].

Most of these studies reported exclusively cross-sectional OCT of the retina data. Resembling our design, Santos et al. reported longitudinal OCT of the retina and Aβ-PET data over 27 months [49]. A decrease in macular RNFL volume was demonstrated in the preclinical AD group (*n* = 15) compared to controls (*n* = 41) and this change was related to Aβ deposition, while GCL volume change was related to age but not to amyloid burden.

Our study design did not allow us to explain the underlying pathological changes of the macular thickening observed in Aβ+ individuals, as we could not directly assess in vivo the presence of Aβ in the retina, we did not have other AD-related biomarkers apart from FBB-PET, neither we had autopsy data available. However, we can speculate that a plausible explanation for the initial macular thickening in Aβ+ individuals could be the accumulation of amyloid deposits in the retina, as it has been reported in AD transgenic mouse models [18], post-mortem human AD brain tissue [13] and using in vivo fluorescent imaging on human retinas from AD patients [47].

Another explanation could be the presence of neuroinflammation, which is an uncontrolled microglia and astroglia activation in the brain in response to failures in homeostasis and tissue damage related to Aβ and tau deposition. Neuroinflammation takes place in the retina as well as in the brain and results in hypertrophy of neurons and glial cells [50]. While the RNFL is made up largely of axons, the macula mostly contains the cell somas [51]. Thus, the oedema secondary to glial cell activation in the macula could account for the selective thickening in this retinal region observed in the SCD Aβ+ group.

Raw OCT retinal data additionally showed that Aβ+ participants experienced thinning of the peripapillary RNFL at v2, although differences did not reach statistical significance after accounting for covariates. This finding could be due to the onset of atrophy in the optic nerve after 24 months in our cohort.

Over 24 months, 15 participants who were initially diagnosed as SCD converted to MCI. It is important to mention that in our study a diagnosis of MCI only required performing under normality in one of the N-BACE battery subtests [23]. Not all the MCI converters experienced memory impairment neither the suspected underlying aetiology for their cognitive deficits was AD. Besides, a few of them returned to a SCD status in the next follow-up study visit (v3), pointing to non-degenerative MCI causes, such as psychological disorders. In line with that, and not unexpectedly, the MCI converter status did not perfectly overlap with PET status (only 5 and 6 of MCI converters were PET+ at v0 and v2, respectively). Probably due to these reasons, we could not detect retinal thickness differences between SCD and MCI participants at v2. In this regard, our data point to amyloid accumulation being a closer endophenotype to the underlying degeneration occurring in very early stages of AD, when cognitive changes are still very subtle. Moreover, the neuropsychological battery used in this study [23] is optimized to detect a more profound cognitive deterioration later in time, but it might be insensitive to the cognitive changes that occur during the prodromal stages of AD.

We acknowledge that our study has several strengths and limitations. The strengths include the use of biomarkers to identify preclinical AD, the detailed characterization of the study participants and the use of age, gender, education, OCT retinal image quality and APOE ε4 as covariates. Limitations include the relatively small sample size (especially for the subgroup with abnormal Aβ), the short follow-up period, the use of PET imaging instead of CSF to determine early Aβ positivity, the use of a convenience sample derived from a research study instead of a population-based one and the exclusion of a substantial part of the cohort due to ophthalmological pathologies known to interfere with retinal thickness measurements.

## Conclusions

In our sample of 129 individuals with SCD, thickening of the inner nasal macular region was associated with FBB-PET+ status and positively correlated with global SUVR. Our data suggest that structural retinal changes are already present in very early stages of the AD continuum and OCT of the retina has potential as a biomarker for preclinical AD. Further research on retinal structural and vascular changes in the AD continuum and their association with amyloid, tau and inflammation biomarkers in larger samples is warranted.

## Supplementary information

**Additional file 1. **Differences between excluded and included participants. Table with demographical, clinical, FBB-PET and OCT differences between those 71 participants excluded from the study and those 129 included in the final sample. A T-test was used to analyse differences on age, years of education, MMSE scores, global SUVR at v0 and all OCT-derived thickness measurements between groups. A Chi-Square test was employed to analyse differences on the distribution of females, APOE genotype, FBB-PET+ at v0 and converters to MCI at v2 between groups. *Statistical significance was set-up at *p* < 0.05. Abbreviations: APOE = apolipoprotein E; MMSE = mini-mental state examination; PET = positron emission tomography; MCI = mild cognitive impairment; v0 = baseline visit; v2 = 2y follow-up visit.

**Additional file 2. **Differences between participants coming from the Memory Clinic and those from the Open House Initiative. Description: Demographical, clinical and FBB-PET differences between participants coming from the Memory Clinic and those from the Open House Initiative. A T-test was used to analyse differences on age, years of education, MMSE scores, and global SUVR at v0. A Chi-Square test was employed to analyse differences on the distribution of females, APOE genotype, FBB-PET+ at v0 and converters to MCI at v2 between groups. *Statistical significance was set-up at *p* < 0.05. Abbreviations: APOE = apolipoprotein E; MMSE = mini-mental state examination; MCI = mild cognitive impairment; OHI = Open House Initiative; v0 = baseline visit; v2 = 2y follow-up visit.

**Additional file 3.** Matrix of correlations of retinal thickness in multiple retinal regions. Description: Values over the diagonal line represent raw Peason’s r correlations between retinal measurements. Values below the diagonal line represent Pearson’s r correlations between retinal measurements adjusted by age, gender, years of education, APOE status and OCT image quality. Statistical significance was set-up at p < 0.05* and < 0.01**. Abbreviations: ETDRS = Early Treatment for Diabetes Retinopathy Study; GCL = ganglion cell layer; RNFL = retinal nerve fiber layer.

## Data Availability

The data, which support this study, are not publicly available but are available from the corresponding author on reasonable request.
