# Supplementary material for: Association between retinal thickness and β-amyloid brain accumulation in individuals with subjective cognitive decline: Fundació ACE Healthy Brain Initiative
Source: Alzheimers Res Ther. 2020 Mar 31;12:37. doi: 10.1186/s13195-020-00602-9 (PMC7110730; doi:10.1186/s13195-020-00602-9)
Supplement: Supplementary file 1 — Additional file 1. Differences between excluded and included participants. Table with demographical, clinical, FBB-PET and OCT differences between those 71 participants excluded from the study and those 129 included in the final sample. A T-test was used to analyse differences on age, years of education, MMSE scores, global SUVR at v0 and all OCT-derived thickness measurements between groups. A Chi-Square test was employed to analyse differences on the distribution of females, APOE genotype, FBB-PET+ at v0 and converters to MCI at v2 between groups. *Statistical significance was set-up at p < 0.05. Abbreviations: APOE = apolipoprotein E; MMSE = mini-mental state examination; PET = positron emission tomography; MCI = mild cognitive impairment; v0 = baseline visit; v2 = 2y follow-up visit. [file 13195_2020_602_MOESM1_ESM.pdf]

Additional file 1.

	**Excluded**	**Included**	**p**
n	71	129	N/A
Age (years)	67,36±7.20	64.72±7.27	0.01*
Females (%)	45 (63.3%)	81 (62.7%)	0.93
Years of education	11.93±4.01	12.49±3.94	0.34
MMSE score	28.99±0.99	29.35±0.88	0.008*
APOE ε4+ (%)	16 (23%)	35 (27%)	0.32
MCI converters at v2	7 (9.86%)	15 (11.63%)	0.70
FBB global SUVR t v0	1.24±0.15	1.22±0.15	0.47
FBB-PET + at v0	10 (14%)	15 (11.63%)	0.62
Peripapillary RNFL			
Total	96,89±18,075	100,29±12,90	0.13
Temporal	72,58±15,83	74,04±11,22	0.45
Superior	114,20±29,29	117,17±22,05	0.43
Nasal	76,04±20,27	77,19±16,43	0.67
Inferior	77,19±16,43	132,74±18,01	0.008*
ETDRS macula			
Center	251,86±41,27	250,86±21,97	0.85
Inner temporal	298,96±32,27	300,65±15,74	0.69
Inner superior	311.00±37,09	312,86±14,98	0.69
Inner nasal	311,66±38,06	314,33±14,84	0.58
Inner inferior	310,39±14,96	307,75±33,85	0.54
Outer temporal	253,87±14,42	251,74±26,71	0.54
Outer superior	268,34±36,14	269,29±13,35	0.84
Outer nasal	283,29±32,62	286,18±14,87	0.49
Outer inferior	256,45±23,46	258,98±14,37	0.42
Macular multilayer			
GCL+	64,59±10,95	64,07±4,84	0.71
RNFL	37,18±8,31	38,18±4,52	0.35
