# Supplementary material for: Association between retinal thickness and β-amyloid brain accumulation in individuals with subjective cognitive decline: Fundació ACE Healthy Brain Initiative
Source: Alzheimers Res Ther. 2020 Mar 31;12:37. doi: 10.1186/s13195-020-00602-9 (PMC7110730; doi:10.1186/s13195-020-00602-9)
Supplement: Supplementary file 2 — Additional file 2. Differences between participants coming from the Memory Clinic and those from the Open House Initiative. Description: Demographical, clinical and FBB-PET differences between participants coming from the Memory Clinic and those from the Open House Initiative. A T-test was used to analyse differences on age, years of education, MMSE scores, and global SUVR at v0. A Chi-Square test was employed to analyse differences on the distribution of females, APOE genotype, FBB-PET+ at v0 and converters to MCI at v2 between groups. *Statistical significance was set-up at p < 0.05. Abbreviations: APOE = apolipoprotein E; MMSE = mini-mental state examination; MCI = mild cognitive impairment; OHI = Open House Initiative; v0 = baseline visit; v2 = 2y follow-up visit. [file 13195_2020_602_MOESM2_ESM.pdf]

Additional file 2.

	**OHI**	**Memory Clinic**	**p**
n	90	39	N/A
Age, years	63.77±7.99	66.89±6.76	0.025*
Females (%)	57 (63.3%)	24 (61.5%)	0.85
Years of education	12,68±3,94	12,04±3,97	0.40
MMSE scores	29,37±0,88	29,31±0,89	0.73
APOE ε4+ (%)	22 (24.4%)	10 (26.6%)	0.89
FBB global SUVR at v0	1.20±0.10	1.27±0.23	0.06
MCI converters at v2 (%)	6 (6.67%)	30 (23.01%)	0.008*
