# Supplementary material for: Association between retinal thickness and β-amyloid brain accumulation in individuals with subjective cognitive decline: Fundació ACE Healthy Brain Initiative
Source: Alzheimers Res Ther. 2020 Mar 31;12:37. doi: 10.1186/s13195-020-00602-9 (PMC7110730; doi:10.1186/s13195-020-00602-9)
Supplement: Supplementary file 3 — Additional file 3. Matrix of correlations of retinal thickness in multiple retinal regions. Description: Values over the diagonal line represent raw Peason’s r correlations between retinal measurements. Values below the diagonal line represent Pearson’s r correlations between retinal measurements adjusted by age, gender, years of education, APOE status and OCT image quality. Statistical significance was set-up at p < 0.05* and < 0.01**. Abbreviations: ETDRS = Early Treatment for Diabetes Retinopathy Study; GCL = ganglion cell layer; RNFL = retinal nerve fiber layer. [file 13195_2020_602_MOESM3_ESM.pdf]

# Additional file 3.

| | Disc total | Disc temporal | Disc superior | Disc nasal | Disc inferior | ETDRS center | ETDRS inner temporal | ETDRS inner superior | ETDRS inner nasal | ETRDS inner inferior | ETRDS outer temporal | ETDRS outer superior | ETDRS outer nasal | ETDRS outer inferior | Macula GCL+ | Macula RNFL |
|---|---|---|---|---|---|---|---|---|---|---|---|---|---|---|---|---|
| **Disc total** | 1 | .411** | .869** | .775** | .810** | .114 | .146 | .143 | .082 | .131 | .192* | .268** | .198** | .223* | .450* | .291** |
| **Disc temporal** | .314** | 1 | .206* | -.032 | .181* | -.007 | -.018 | .028 | -.062 | -.021 | .008 | .114 | .097 | .089 | .211* | .477** |
| **Disc superior** | .870** | .192* | 1 | .581** | .571** | .045 | .167 | .163 | .121 | .173* | .168 | .237** | .173 | .185* | .341** | .247** |
| **Disc nasal** | .770** | -.114 | .599** | 1 | .570** | .155 | .112 | .082 | .048 | .049 | .147 | .164 | .126 | .096 | .256** | .169 |
| **Disc inferior** | .834** | .146 | .571** | .563** | 1 | .204* | .212* | .235** | .211* | .228** | .270** | .355** | .284** | .349** | .566** | .134 |
| **ETDRS center** | .196** | .068 | .052 | .194* | .250** | 1 | .462** | .399** | .590** | .330** | .202* | .240** | .224* | .312** | .254** | .310** |
| **ETDRS inner temporal** | .232** | .056 | .182* | .146 | .255** | .435** | 1 | .899** | .879** | .859** | .463** | .644** | .531** | .482** | .339** | .172** |
| **ETDRS inner superior** | .242** | .116 | .169 | .127 | .277** | .374** | .899** | 1 | .896** | .898** | .541** | .778** | .690** | .473** | .439** | .203* |
| **ETDRS inner nasal** | .187* | .030 | .128 | .097 | .252 | .579** | .884** | .893** | 1 | .875** | .439** | .636** | .643** | .515** | .401** | .227** |
| **ETDRS inner inferior** | .207* | .046 | .182* | .078 | .257** | .316** | .870** | .901** | .876** | 1 | .517** | .678** | .681** | .552** | .396** | .143 |
| **ETDRS outer temporal** | .220* | .013 | .138 | .178* | .271** | .204* | .479** | .552** | .446** | .532** | 1 | .687** | .597** | .597** | .491** | .154 |
| **ETRDS outer superior** | .331** | .170 | .232** | .192* | .357** | .246** | .663** | .797** | .643** | .697** | .691** | 1 | .804** | .676** | .575** | .294** |
| **ETDRS outer nasal** | .261** | .153 | .160** | .163 | .285** | .239** | .564** | .711** | .653** | .692** | .597** | .808** | 1 | .718** | .595** | .246** |
| **ETDRS outer inferior** | .265** | .148 | .173 | .106 | .336** | .343** | .522** | .495** | .530** | .571** | .602** | .665** | .709** | 1 | .522** | .279** |
| **Macula GCL+** | .498** | .263** | .319** | .276** | .575** | .292** | .399** | .486** | .439** | .422** | .481** | .602** | .595** | .497** | 1 | .221** |
| **Macula RNFL** | .332** | .524** | .247** | .191* | .135 | .331** | .191* | .213* | .235** | .147 | .154 | .298** | .241** | .283** | .231* | 1 |
